# Adverse effects of biologic anti-inflammatory agents on the respiratory system: A review

**DOI:** 10.7196/AJTCCM.2021.v27i2.117

**Published:** 2021-06-23

**Authors:** D Joseph, G R Tintinger, J A Ker, N Pannell

**Affiliations:** Department of Internal Medicine, School of Medicine, Faculty of Health Sciences, University of Pretoria, Pretoria, South Africa

**Keywords:** biologic anti-inflammatory therapy, side-effects, adverse reactions, lung disease, rheumatological conditions

## Abstract

The therapy of autoimmune rheumatological conditions has undergone significant changes with the introduction of biologic antiinflammatory agents including cytokine antagonists and agents that interfere with the function of T and B cells or those that inhibit intracellular enzymes such as Janus kinase (JAK). Although useful to control inflammation, these agents may be associated with druginduced lung disease, which may be difficult to differentiate from pulmonary disorders caused by the underlying autoimmune diseases. This review aims to provide a description of lung disease, both infectious and non-infectious, that may be induced by the administration of biologic anti-inflammatory agents with emphasis on inhibitors of tumour necrosis factor, interleukin-1, interleukin-6 and JAK.

## Background

The recent introduction of an array of biologic anti-inflammatory agents has transformed the treatment of numerous rheumatological conditions and other autoimmune disorders such as inflammatory bowel disease and psoriasis. However, despite important clinical benefits, these biologic agents may be associated with adverse effects involving multiple systems, including the respiratory tract. There is increasing evidence that these biologics may paradoxically induce an autoimmune process and this can be as part of a systemic process such as the induction of systemic lupus erythematosus, vasculitis, sarcoidosis and antiphospholipid syndrome. There is also an association between these drugs and organ-specific auto-immune processes such as interstitial lung disease, uveitis, optic neuritis, peripheral neuropathies, multiple sclerosis and auto-immune hepatitis.

The wider use of biological agents also requires constant awareness of the risks associated with these agents by the treating physicians as experience is gained by using these drugs

Lung involvement in patients with rheumatological disorders is common and a wide spectrum of abnormalities may occur. Respiratory physicians need to differentiate lung disease associated with the underlying rheumatological condition from adverse effects secondary to various forms of therapy. It is important to recognise potential side-effects of therapeutic agents to facilitate the diagnostic process. This review will focus on the adverse effects of the most commonly used anti-inflammatory biologic disease-modifying agents on the respiratory system. These agents have been selected and classified according to their predominant mode of action. Biologic agents are categorised into four classes based on their specific targets: (i) inhibitors of cytokines; (ii) agents targeting signal transduction pathways; (iii) interference with intercellular interactions; and (iv) depletion of inflammatory cells.

The classes of biologic agents as well as examples of each are shown in Table 1.

**Table 1:** Classification of biologic anti-inflammatory agents

**Class of agent based on** **mechanism of action**	**Examples**
**1. Inhibition of cytokines:**	
TNF-α	Etanercept, infliximab, adalimumab, certolizumab, golimumab
IL-1	Anakinra, canakinumab, rilonacept
IL-6	Tocilizumab
**2. Interference with signal transduction pathways by inhibition of tyrosine kinases or JAK**	Tofacitinib, baricitinib, upadacitinib, filgotinib, peficitinib
**3. Inhibition of intercellular adhesion**	Abatacept
**4. Depletion of B cells**	Rituximab

TNF-a = tumour necrosis factor- alpha

IL = interleukin

JAK = Janus kinase

## Cytokine antagonists

Cytokines are small, short-lived protein molecules, which function as important mediators of intercellular communication required for an integrated response of immune and inflammatory processes.^[1]^ With novel applications of highly specific cytokine antagonism, considerable knowledge is being gained about the biology of these molecules, as well as the impact these targeted therapies may have as treatment options for a variety of inflammatory diseases.^[2]^

The adverse effects of the well-established classes of cytokine inhibitor therapies focusing on tumour necrosis factor (TNF) inhibitors, as well as the interleukin 1 (IL-1) and interleukin 6 (IL6) inhibitors, are described below.

### Tumour necrosis factor inhibitors

Tumour necrosis factor-alpha (TNF-α) is produced primarily by macrophages in response to inflammatory stimuli, and binds to its receptors that are found on almost all cells in the body, resulting in signalling required for normal development and functioning of the immune system.^[3]^ TNF-α has a broad spectrum of biological activity and there is considerable evidence showing that either overproduction or inadequate production plays an important role in the pathogenesis of various chronic inflammatory diseases.^[3]^

TNF-α exists in two forms: a soluble form and as a precursor transmembrane form, which is also biologically active in cell-to-cell interactions.^[4]^ Both forms of TNF-α interact with two types of receptors, which are expressed on almost all nucleated cells: TNF receptor 1 (TNFRI), a 55 kDa receptor also known as the p55 receptor and TNF receptor 2 (TNFRII), a 75 kDa receptor also known as the p75 receptor.^[3]^ Binding of TNF-α to these receptors culminates in a variety of downstream effects including the stimulation of apoptosis, various anti-tumour responses and multiple effects on the immune system development and functioning.^[3–6]^

The preferential receptor for transmembrane TNF-α appears to be TNFRII, which has both shared and opposing effects compared with those of TNFRI. The differential effect of the various TNF-α inhibitors, particularly on the transmembrane form of TNF-α, may account for the differences in clinical effect, as well as adverse risk profiles of the different agents.^[4]^ Five TNF-α inhibitors are available and have been extensively studied: etanercept, infliximab, adalimumab, certolizumab and golimumab.

Etanercept is a soluble recombinant human TNF-α dimeric fusion protein comprising the extracellular portion of two p75 TNF receptors fused to the Fc portion of immunoglobulin G (IgG).^[6,7]^ In circulation, etanercept will bind sTNF-α and prevent it from interacting with the TNF receptors, resulting in TNF-α antagonism.

Infliximab is a monoclonal antibody (mAb) that neutralises the biological activity of TNF-α. It is a chimeric antibody comprising both murine and human portions.^[6,7]^ In contrast, adalimumab was the first fully human mAb against TNF-α to enter clinical trials and as such is less prone to the risk of the development of antidrug antibodies, enabling more long-term administration.^[7,8]^

Both certolizumab (a humanised anti-TNF antibody Fab’ fragment chemically linked to propylene glycol) and golimumab (an IgG1 kappa mAb specific for human TNF-α) were developed later and have shown efficacy in neutralising both transmembrane and solubleTNF-α.^[9–11]^

The adverse effects of TNF-α antagonism involving the respiratory system are numerous and may be of a serious nature. These include both infectious and non-infectious complications.

The role of TNF-α in the normal immune response to infection is well described and infectious complications resulting from inhibitors of TNF are therefore to be expected. Evaluating the risk of infection from these agents is difficult, given that they are used in patients who are already immunocompromised from the underlying disease itself or the effects of prior or concomitant immunosuppressive therapy.^[12]^

Infections, especially those involving the respiratory tract, are however frequent problems encountered in patients using TNF-α inhibitors.^[13]^ The infection rates appear higher in patients receiving mAbs compared with etanercept and there appears to be a higher rate of infection in patients receiving higher doses.^[12]^ Both common and opportunistic infections have been described.^[14,15]^ The infection risk appears to decrease over time and is highest in the first 6 months after initiation of therapy.^[12]^ Data appear to be conflicting regarding the rate of serious infections associated with TNF-α inhibitors, with some studies reporting no increased serious infection rate compared with controls, while others show a tendency for serious infections to occur in line with what is known about the biological role of TNF-α in infection control.^[14–16]^

Owing to the critical role TNF-α plays in the control of tuberculosis, the impact of its inhibition, particularly with the mAbs, on reactivation of tuberculosis is an important consideration.^[17–18]^ TNF-α inhibitors have been associated with increased susceptibility to tuberculosis infection, poor granuloma formation, greater rates of tuberculosis reactivation and higher mortality from tuberculosis.^[15,17,18]^ Screening for latent tuberculosis is recommended prior to initiating these agents. However, the possibility of cutaneous anergy in patients with underlying autoimmune disease must also be considered.^[15,18]^ A higher proportion of patients receiving TNF-α inhibitors experience extrapulmonary or disseminated tuberculosis compared with infections in the general population, suggesting that this may be a class effect.^[15,17,18]^

Infliximab and adalimumab are effective in the management of various non-infectious granulomatous diseases including sarcoidosis, granulomatosis with polyangiitis and Crohn’s disease.^[4,19,20]^ Etanercept has been shown to be less effective in the management of these conditions. The risk for tuberculosis appears to be lower with etanercept compared with the use of the mAb forms of TNF inhibition, suggesting that the greater the antigranuloma forming effect, the greater the risk for granulomatous infections.^[4]^ The differential effects of these agents are likely mediated through varying efficacy in eliminating immune cells bearing transmembrane TNF-α.^[4]^

TNF inhibitors have been shown to be effective in treating both the underlying inflammatory disease for which they are being used, as well as providing benefit for any associated interstitial lung disease (ILD). Paradoxically however, these agents themselves may be associated with new-onset ILD, as well as exacerbations of pre-existing ILD.^[21,22]^

The predominant pattern of ILD involvement is a usual interstitial pneumonia (UIP) pattern, which has a high mortality in patients with prior UIP.^[23]^ Other patterns that have been described include organising pneumonia, nonspecific interstitial pneumonia (NSIP), diffuse alveolar haemorrhage (DAH) and lymphocytic interstitial pneumonitis (LIP).^[21,23]^ Studies describing these findings have however been criticised for not conclusively demonstrating an absence of ILD prior to initiation of TNF inhibitors and many patients using these agents did so in conjunction with other disease-modifying antirheumatic drugs (DMARDs) known to produce similar effects in the lung.^[23]^

According to data obtained from the British Society of Rheumatology Biologics Register, the mortality rate from ILD in patients with rheumatoid arthritis (RA) treated with TNF-α inhibitors was similar to those treated with conventional DMARDs.^[22]^ A recent review by Huang *et al.*^[24]^ highlighted that the use of TNF-α inhibitors in patients known to have RA-associated ILD may predispose them to more severe symptoms and even death.^[24]^

While TNF-α inhibition has been used successfully to treat noninfectious granulomatous diseases, paradoxically TNF-α antagonism has also been associated with the development of a granulomatous, sarcoid-like disease, which may have both pulmonary and extra-pulmonary effects.^[21,23,25]^ In almost all instances, the disease resolves on withdrawal of therapy, providing compelling evidence of causality.^[23,25]^ The presence of pulmonary granulomas could also be explained by the development of sarcoidosis, hypersensitivity pneumonitis or an unidentified infectious agent, although the latter is unlikely given the continued resolution of disease despite initiation of replacement immunosuppressive regimens after withdrawal of TNF-α inhibitors.^[13]^

The proposed mechanisms for these granulomatous changes include an enhanced pro-inflammatory and pro-fibrotic role of interferon gamma (IFN-γ) and IL-1 in the absence of TNF-α.^[26]^ This results in an imbalance of the TNF-α-IFN-γ immunoregulatory pathway, which may be responsible for the subsequent development of granulomatous change.^[23]^ This could be explained by the differences in their pharmacokinetic and pharmacodynamic properties, as well as their effect on transmembrane TNF-α bearing cells.^[25,27]^

Accelerated pulmonary rheumatoid nodule formation has been described in patients receiving etanercept. These patients simultaneously experienced reduced joint symptoms, and resolution or stabilisation was demonstrated on withdrawal of etanercept, again suggesting causality.^[13,23]^

It has been suggested that the use of TNF-α inhibitors may be associated with an increased risk of lymphoproliferative disorders, especially non-Hodgkin’s lymphoma in patients with RA. It has been shown that the rate of lymphoma is increased in patients with severe and active RA, the very group of patients that are more likely to receive these agents.^[15]^ TNF-α is also known to modulate tumour development and spread; however, cancers arising during therapy with TNF-α inhibitors were not shown to differ by stage at presentation or post-cancer survival rates. ^[28]^

There is a growing body of literature suggesting that TNF-α antagonists may induce autoimmune diseases, although the appearance of new autoantibodies during therapy is not clearly understood. Most patients who develop autoantibodies remain asymptomatic. However, autoimmune disease may manifest clinically with vasculitis, the antisynthetase syndrome, and lupus-like syndrome involving the lung and pleura.^[13,23]^ Studies have also demonstrated induction of antiphospholipid antibodies, but thrombotic events have not been reported in these patients.^[23]^

It should be noted that in addition to the adverse respiratory effects associated with antagonism of TNF-α, these agents have also been used in clinical trials for treating various respiratory diseases including asthma, chronic obstructive pulmonary disease, sarcoidosis and pulmonary fibrosis. Studies have thus far yielded inconsistent results.^[23,29]^

### Interleukin-1 inhibitors

IL-1 does not refer to a single cytokine, but rather encompasses a family of molecules, which have an important role in mediating the inflammatory response, including the induction of IL-6.^[7,30]^ IL-1 cytokines are known to function as potent inflammatory mediators, and play a role in normal immune system function, and immune-mediated disease, including cardiovascular, metabolic, neuro-degenerative and neoplastic diseases.^[30]^ Of particular importance is IL-1-beta (IL-1β), which is an important target for IL-1 antagonism.^[31]^

The three commercially available IL-1 inhibitors are anakinra, canakinumab and rilonacept.

Anakinra is a recombinant IL-1 receptor antagonist that binds to IL-1 receptor type 1 (IL-1R1), which is the target receptor for IL-1α and IL-1β.^[7]^ It is structurally similar to endogenous IL-1 receptor antagonist (IL-1Ra), which is a naturally occurring competitive IL-1R1 antagonist. Apart from its role in the management of auto-inflammatory diseases, it has also been investigated for the management of COVID-19-associated pulmonary complications.^[32]^

Canakinumab is a human mAb targeting IL-1β, and it was developed for the treatment of immune disorders including various forms of inflammatory arthritis such as adult-onset Still’s disease and RA.^[33]^

Rilonacept is a synthetic protein containing the extracellular domains of IL-1R1 that behaves as a soluble decoy receptor for IL-1β, and binds with high affinity.^[34]^ It is currently used for the treatment of cryopyrin-associated periodic syndromes (CAPS) in adults and children 12 years and older.

The most serious adverse effect of the IL-1 inhibitors appears to be an increased risk of infection.^[7]^ The risk is surprisingly modest and is likely influenced by additional therapies the patient may be using, the dose used, as well as other underlying co-morbid disease.^[15,35-37]^ Most notably, the presence of asthma is associated with a greater risk of pneumonia and anakinra should be used with caution in asthmatics.^[15,35]^ Reversible neutropenia has also been described and may contribute to the risk of infection.^[7,38]^

A meta-analysis of anakinra conducted in 2009 showed that there was an insignificant increase of infections in patients using anakinra v. placebo, including opportunistic infections such as tuberculosis.^[39]^ The Canakinumab Anti-inflammatory Thrombosis Outcome Study (CANTOS) trial,^[40]^ which examined the role of anti-inflammatory therapy during atherosclerosis, found a reduction in the risk of cardiovascular events and this was countered by an increase in death due to infections (incidence rate in pooled canakinumab group was 0.31 events per 100 person-years v. 0.18 events per 100 person-years in the placebo group). In addition, the increased risk of infection was largely found in older patients or those with diabetes.

Anakinra should not be initiated in patients with active infection and should be discontinued in patients who develop severe infection. Caution should also be exercised prior to initiating therapy in patients with a history of chronic or recurrent infection. The concomitant use of TNF-α antagonists may increase the risk of infection and should be avoided.^[7,41]^

Anakinra has been associated with drug-induced ILD and pulmonary fibrosis when used in patients with RA.^[42]^ A severe inflammatory ILD associated with a high mortality has emerged in patients taking IL-1 and IL-6 inhibitors for the management of systemic juvenile idiopathic arthritis, suggesting a causal link with these agents, although the exact relationship remains unknown.^[43–45]^

### Interleukin-6 inhibitors

IL-6 is a pleiotropic cytokine with multiple biological effects, and plays an important role in many immune and inflammatory processes.^[46]^ Tocilizumab is an anti-human IL-6 receptor antibody, which binds to the IL-6 receptor (IL-6R), thereby interfering with the effects of cytokine binding. It has been shown to be effective in the treatment of RA.^[47,48]^ It is also used in the management of juvenile idiopathic arthritis, giant cell arteritis and the cytokine release syndrome. In addition to anakinra, tocilizumab is currently being investigated for the management of COVID-19-associated pulmonary complications.^[49]^

Tocilizumab has a good long-term safety profile,^[50,51]^ but various adverse effects have been attributed to it including infections, cytopenia and neutropenia, hyperlipidaemia and abnormal liver enzymes.^[50,52,54]^ The neutropenia associated with IL-6R inhibition does not appear to be associated with a risk of severe infection.^[51,52]^

The respiratory effects of tocilizumab are mainly mild in nature with upper respiratory tract infections, bronchitis, cough, dyspnoea and nasopharyngitis described.^[50]^ The USA^[55]^ has issued black box warnings regarding infection risk associated with the use of tocilizumab, noting an increased risk of serious and potentially fatal infections, as well as an increased risk of tuberculosis (pulmonary and extra-pulmonary). Tocilizumab may in addition mask the symptoms of infection and care should be taken not to overlook a patient presenting with mild symptoms.^[52]^

Randomised control trials evaluating the use of tocilizumab in RA have revealed non-infectious pulmonary adverse effects in a minority of patients.^[53,54,56]^ Most of these occurred in patients on concomitant DMARDs therapy. Conditions described include new-onset ILD, idiopathic pulmonary fibrosis and allergic pneumonitis. Severe exacerbation of RA-associated ILD has also been described.^[53,56]^ Case reports have been published describing organising pneumonia, acute pneumonitis, as well as exacerbation of combined pulmonary fibrosis and emphysema syndrome associated with use of tocilizumab.^[57–59]^

## Interference with signal transduction pathways: tyrosine kinase inhibitors

Tyrosine kinases are intracellular enzymes that transmit signals by transferring phosphate groups to tyrosine residues on cytoplasmic proteins or the intracellular domains of transmembrane receptors.^[60–61]^ They are classified as targeted synthetic DMARDs.^[62]^

The mechanism of action of Janus kinase (JAK) inhibitors is based on inhibition of activity of one or more JAK isoforms, thus impeding certain pathways, particularly the JAK-signal transducer and activator of transcription (JAK-STAT) pathway.^[63]^ There are four JAK isoforms in humans: JAK1, JAK2, JAK3 and tyrosine kinase 2 (TYK2), which function in pairs and whose activity is related to different cytokine receptors.^[64]^

The first JAK inhibitor approved by the FDA for RA was tofacitinib, which inhibits JAK1 and JAK3 resulting in the inhibition of IFN-γ and IL- 6.^[64,65]^

Tofacitinib is indicated for the treatment of adult patients with moderate to severe active RA, who have had an inadequate response or intolerance to methotrexate. It may be used as monotherapy or in combination with methotrexate or other non-biologic DMARDs. Use in combination with biologic DMARDs or potent immunosuppressants such as azathioprine or cyclosporine is not recommended.

During the ORAL sequel long-term extension study that evaluated the efficacy and safety of tofacitinib 5 mg and 10 mg twice daily, the observed incidence rates for serious infections, deep vein thrombosis and pulmonary embolism were similar to those seen with TNF inhibitors and other biologic DMARDs.^[66]^ The most frequently reported adverse events that led to temporary discontinuation or dose reduction of tofacitinib were nasopharyngitis and herpes zoster.^[67,68]^ The reactivation of varicella-zoster virus is a concern with the use of tofacitinib as disseminated disease may be fatal and vaccination should be considered prior to the initiation of biologic therapy in patients with RA older than 60 years.^[69]^

Patients with RA are at greater risk of serious infections, including tuberculosis.^[68]^ The risk of tuberculosis in patients receiving tofacitinib treatment varies depending on the prevalence of tuberculosis in different geographic areas. Incidence rates are comparable with data for biologic DMARDs^[66]^ and screening for latent tuberculosis is advised prior to starting treatment with tofacitinib.

The FDA issued a warning regarding serious infections leading to hospitalisation or death with the use of tofacitinib. The most common serious respiratory infection was pneumonia^[70]^ with tuberculosis and other mycobacterial infections, cryptococcosis, histoplasmosis, pneumocystis, cytomegalovirus, BK virus infection and listeriosis have also been reported.

Baricitinib is a selective JAK1/JAK2 inhibitor^[71]^ that shares the same infection risks as tofacitinib. One particular safety concern was the possible increased risk of thromboembolic events related to the use of baricitinib.^[72]^ The European Medicine Agency (EMA) has issued warnings to use baricitinib and tofacitinib with caution in patients with risk factors for thromboembolic disease.^[73]^

Upadacitinib is a selective JAK1 inhibitor,^[74]^ and has demonstrated a favourable risk-benefit ratio in patients with an inadequate response to biologic DMARDs at a dose of 15 mg daily.^[74]^ The FDA issued a warning for upadacitinib similar to that for baricitinib and venous thromboembolic events occurred in patients with additional risk factors.^[75]^

## Inhibition of intercellular adhesion

Antigen presenting cells (APCs) such as dendritic cells and macrophages present antigens to T cells, priming their activation. Optimal activation of T cells requires a co-stimulatory signal provided by the interaction between CD28 on the surface of T cells and CD80/CD86 on APCs.^[76]^ Abatacept, a homologue of CD28, competitively binds to CD80/CD86 resulting in suppression of T cell activation.^[76]^ Pulmonary hypertension and acute respiratory failure have been reported in patients receiving abatacept.^[77,78]^

## Depletion of B cells

Rituximab is an anti-CD20 monoclonal antibody that targets B cells, causing their depletion via multiple mechanisms including complement-mediated and antibody-dependent cytotoxicity.^[79]^ Patients receiving rituximab rarely develop an organising pneumonia. Another rare, but potentially fatal, complication that has been reported with rituximab is the new onset of an ILD.^[80]^ This complication appears to be more common in middle-aged males, presents as bilateral infiltrates on computed tomography (CT) imaging of the lungs, and is associated with hypoxaemia and a restrictive deficit on pulmonary function testing.^[80]^ A summary of the adverse effects of biologic agents on the respiratory system are shown in Table 2.

**Table 2:** Different classes of biologic anti-inflammatory therapy and the adverse effects of these agents on the respiratory system

**Class of agent based on**	**Adverse effects on the respiratory system**	
**mechanism of action**	**Infectious**	**Non-infectious**
**1. Inhibition of cytokines**		
TNF-α	TB and other opportunistic infections	UIP, NSIP, LIP, diffuse alveolar haemorrhage, granulomatous lung disease, accelerated rheumatoid nodules, lymphomas, autoimmune disease
IL-1	Pneumonia, TB	Interstitial lung disease
IL-6	URTI, bronchitis, TB	Interstitial lung disease, organising pneumonia, allergic pneumonitis
**2. Interference with signal transduction pathways by inhibition of tyrosine kinases or JAK**	Nasopharyngitis, herpes zoster, TB, pneumonia, including fungal and viral pneumonia	Pulmonary emboli
**3. Inhibition of intracellular adhesion**		Pulmonary hypertension, acute respiratory failure
**4. Depletion of B cells**		Organising pneumonia, acute interstitial pneumonitis

TNFα = tumour necrosis factor-α

TB = tuberculosis

UIP = usual interstitial pneumonia

NSIP = nonspecific interstitial pneumonia

LIP = lymphocytic interstitial pneumonia

IL = interleukin

ILD = interstitial lung disease

URTI = upper respiratory tract infection

JAK = Janus kinase

## Approach to the diagnosis of druginduced lung disease

A chest CT study of 332 patients with RA on long-term biologic therapy showed the following forms of respiratory involvement:^[81]^

1. ILD in 29 patients
2. airway disease in 76 patients which included bronchial wall thickening, bronchiectasis, bronchiolitis, air trapping and atelectasis
3. co-existing interstitial disease with airway disease in 6 patients
4. no CT abnormalities of the lungs in 221 patients.

When evaluating patients with possible drug-induced lung disease, it is important to obtain a thorough history relating to the timing of initiation of medications and the onset of symptoms. Clinical examination may yield signs supporting an allergic reaction, but this is not always the case. Importantly, the respiratory physician may need to differentiate the underlying lung disease from other causes such as infections, malignancy, cardiac failure and adverse effects of drugs used.^[82]^ This usually requires pulmonary function testing, chest radiographs and high-resolution chest CT. ^[82]^ If the diagnosis is still in doubt, a bronchoscopy with lavage and/or transbronchial biopsies should be performed.

## Conclusion

Biologic anti-inflammatory agents used for the treatment of rheumatological disorders may cause respiratory infections and non-infectious pulmonary disease. Respiratory physicians need to be cognisant of the adverse effects of biologic agents when evaluating rheumatology patients with pulmonary abnormalities.
